# Use of Flow Cytometry in Clinical Practice

**DOI:** 10.6004/jadpro.2015.6.5.4

**Published:** 2015-09-01

**Authors:** Dawn M. Betters

**Affiliations:** Case Western Reserve University, Cleveland, Ohio

## ABSTRACT

**Case Study**

Mrs. K is a 63-year-old woman who presented to the clinic with complaints of fatigue, bruising, weight loss of 20 pounds in the past 2 months, and decreased appetite. She stated that she had constant moderately severe joint pain and napped three times a day in an attempt to battle her fatigue. However, the naps resulted in no change in her fatigue. Her increase in fatigue interfered with her daily functioning, and she was unable to enjoy spending time with her family.

Mrs. K’s medical history included breast cancer diagnosed in 2006 and hypertension diagnosed in 2010. For her breast cancer, she was treated with chemotherapy and radiation therapy. She had no other pertinent medical history and no significant family history.

Physical examination revealed temperature of 38.9˚C, heart rate of 108 beats per minute, blood pressure of 158/68, respiratory rate of 22 breaths per minute, oxygen saturation of 96%; skin intact, pale, and cool to touch; scattered petechiae were present over both her lower extremities, abdomen, and back; and pale oral mucosa were observed. All other systems reviewed were unremarkable.

Laboratory workup revealed the following measures: peripheral blood with white blood cell count of 110,000 x 10₃/µL, hemoglobin of 8.6 g/dL, hematocrit of 28%, and a platelet count of 8,000 x 10₃/µL. A bone marrow biopsy evaluated by flow cytometry revealed 22.6% CD34⁺/CD117⁺ blasts (Figure 1).

**Figure 1:**
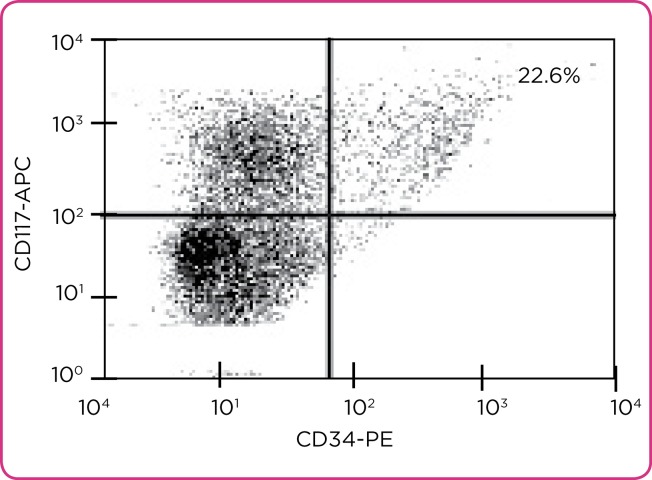
Flow cytometry depiction of blast cells. APC = allophycocyanin; PE = phycoerythrin.

## ARTICLE

For advanced practitioners (APs) working with oncology patients, "22.6% CD34⁺/CD117⁺ blasts" on flow cytometry, as seen in Mrs. K’s case study above, may be familiar, but understanding and interpreting this laboratory result may be more challenging. Flow cytometry is a powerful, well-established method for effectively measuring attributes of cells, including cellular property functions, primarily by using cluster of differentiation (CD) properties of a cell.

The first flow cytometer was constructed roughly 60 years ago (Giaretti, 1997; Jaroszeski & Radcliff, 1999; Picot, Guerin, Le Van Kim, & Boulanger, 2012). The use of flow cytometry has expanded to provide invaluable information for many diseases and conditions (Davis et al., 2007; De Rosa, Brenchley, & Roederer, 2003). The purpose of this article is to provide a brief overview of the methodology of flow cytometry for the AP and to highlight some applications of flow cytometry in clinical practice.

## Brief Overview

Flow cytometry is the process in which cells in an isotonic buffer suspension pass through a laser beam one by one (Macey, 2007). Quantifiable measurements of cellular attributes, such as cell size, granularity, DNA/RNA content, surface and intracellular receptors, and gene expression, are made using the principle of fluorescence, or excited light energy (Macey, 2007; Mach, Thimmesch, Orr, Slusser, & Pierce, 2010). The flow cytometer consists of four systems: fluidics, optics, electronics, and computer interface.

**Figure 2:**
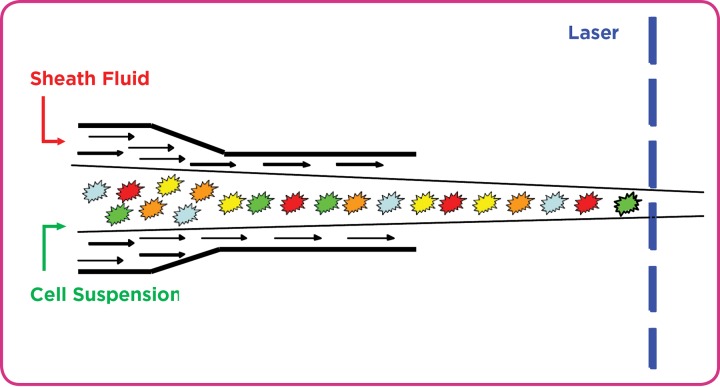
The flow cytometer’s hydrodynamic force, created by sheath fluid, drives cell suspensions through a laser beam–sensing area one at a time.

The first step is fluidics. Cellular suspensions, the most common source of material used in flow cytometry for disease characterization (Ibrahim& van den Engh, 2007) and usually derived from blood, tissue, or tumors, are labeled using fluorochrome-labeled monoclonal antibodies. Antibodies are proteins that recognize and bind to specific antigen structures on the cell surface, or intracellularly, to identify the measurement(s) of interest. Fluorochromes, compounds that emit light when excited, are bound to the antibodies (Ibrahim& van den Engh, 2007; Jaroszeski & Radcliff, 1999; Pedreira, Costa, Lecrevisse, van Dongen, & Orfao, 2013; Picot et al., 2012). The fluorochrome-labeled cells in liquid suspension then pass individually and rapidly through a laser beam–sensing area (Figure 2; Macey, 2007).

Optics consists of the light-amplification source (laser). The laser emits light of a specified wavelength. There are several types of lasers, each capable of exciting specific fluorochromes and causing them to emit light ((Jaroszeski & Radcliff, 1999).

Once the suspension passes the laser, two initial detections are made. Forward scatter (FSC) is light that scatters in the same direction as the laser and is indicative of cellular morphology, or cell size. Side scatter (SSC) is light that is scattered at a 90˚ angle from the direction of the laser and is proportional to cell granularity (structures within the cell), or cell density ((Ibrahim& van den Engh, 2007; (Jaroszeski & Radcliff, 1999; (Macey, 2007; (Mach et al., 2010; (Pedreira et al., 2013; (Picot et al., 2012).

**Figure 3:**
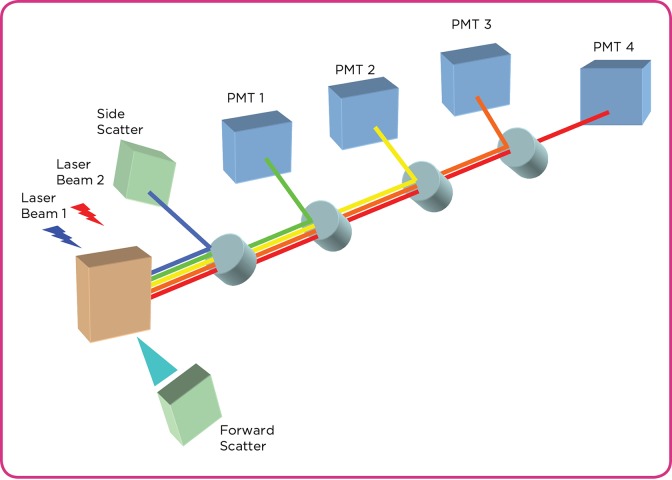
Basic schematic of flow cytometer optical system. PMT = photomultiplier tube.

The electronics involve the conversion of photons into interpretable data. The flow cytometer’s photomultiplier tubes (PMTs) are semiconductors that generate electrical current based on light detection. The PMTs will convert the detected photons into electrical signals that can be interpreted (Figure 3; (Jaroszeski & Radcliff, 1999; (Macey, 2007; (Pedreira et al., 2013; (Picot et al., 2012).

Each signal detected by the cytometer is known as an "event," and all events for a prepared sample are acquired by the cytometer and stored on the computer. The computer is also responsible for regulating the function of the flow cytometer. Graphic analysis of the samples can be interpreted in one-, two-, or three-dimensional images using software designed for flow cytometry data analysis ((Macey, 2007).

**Figure 4:**
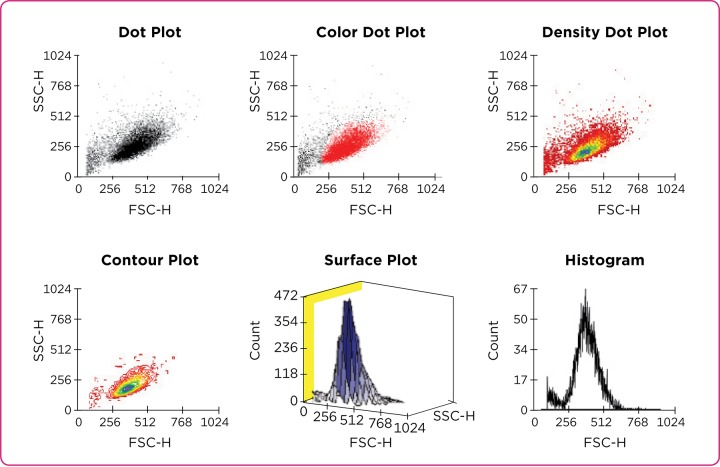
Types of data-analysis plots. SSC-H = side scatter histogram; FSC-H = forward scatter histogram.

Data analysis is often depicted in graphic formats and accompanies patient test results. It can be displayed in a variety of ways. Histograms display one measurement of interest, whereas dot plots display two parameters of interest (Figure 4; (Herzenberg, Tung, Moore, Herzenberg, & Parks, 2006; (Ibrahim& van den Engh, 2007; (Jaroszeski & Radcliff, 1999; (Pedreira et al., 2013).

Many flow cytometers are capable of cell sorting. Cells or cell particles, such as lymphocytes or chromosomes, may be physically separated from the heterogeneous suspension and collected as a highly purified suspension ((Macey, 2007). This process allows for the observation and analysis of a uniform population for investigation. More clinically relevant, it allows for the selection of cells with particular features often used to diagnose or treat a disease or condition ((Koss & Melamed, 2006; (Macey, 2007).

## Applications to Clinical Practice

Flow cytometry is often used to characterize diseases in clinical settings ((Barlogie et al., 1983; (Ibrahim & van den Engh, 2007). Peripheral blood, bone marrow aspirate, and cerebrospinal fluid are all specimens that can be analyzed using flow cytometry. However, only viable cells can be analyzed. If the sample does not contain viable cells, flow cytometry analysis is not an option ((Trewhitt, 2001).

Flow cytometry is most commonly indicated for both benign and malignant hematologic processes. It can aid in several clinical areas, including diagnosis, treatment plans, and monitoring residual or relapsed disease ((Craig & Foon, 2008; (Wood et al., 2007).

Measurement of DNA content was one of the earliest uses of flow cytometry ((Barlogie et al., 1983; (Giaretti, 1997). A 67% increase in DNA content was noted in malignant cells compared with nonmalignant cells ((Barlogie et al., 1983). Normal, healthy cells are diploid (two complete sets of chromosomes), whereas malignant cells most often have abnormalities in their chromosomes, which can be quantified by flow cytometry ((Friedlander, Hedley, & Taylor, 1984). Retrospective studies examined the relationship between DNA abnormalities and survival duration in patients with oncologic disease in an attempt to predict prognosis ((Barlogie et al., 1983; (Merkel, Dressler, & McGuire, 1987).

Phenotyping, the identification of specific observable characteristics, is another common use of flow cytometry in oncology. There are many phenotypic designations to differentiate healthy cells from tumor cells. The Table provides basic CD specification for common immune cell phenotypes ((Maecker, McCoy, & Nussenblatt, 2012).

**Table 1:**
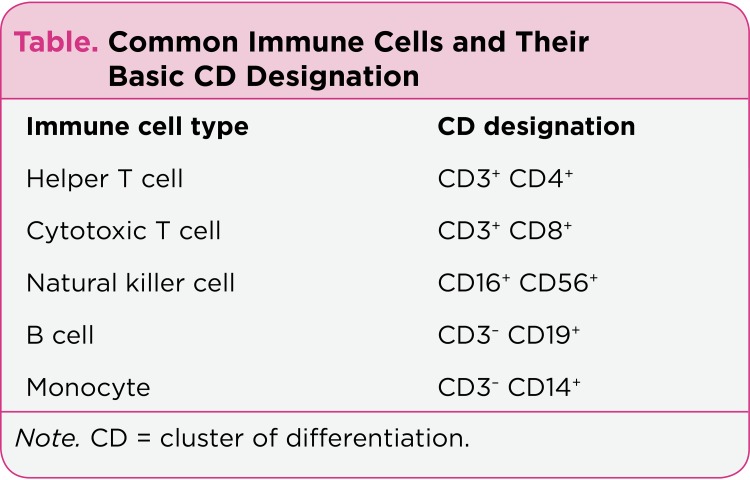
Common Immune Cells and Their Basic CD Designation

Tumor cells often express distinguishable surface receptors. For example, flow cytometry analysis of patients with acute promyelocytic leukemia (APL) has been shown to demonstrate a myeloid phenotype as CD11b⁻ and CD11c⁻. In contrast, in normal myeloid cells, the phenotype is CD11b⁺ and CD11c⁺ ((Dong, Kung, Bhardwaj, & McGill, 2011).

Designation of T-cell or B-cell lineage in acute lymphoblastic leukemias (ALLs) is another example of phenotyping. Once denoted T-cell or B-cell ALL, each lineage can be refined by further immunoprofiling to determine subgroups and prognosis. Varma and Naseem ((2011) have described the phenotype of each subgroup in detail. A pre–B-cell leukemia will express CD19 and CD10, whereas a mature B-cell leukemia will express CD19 but not CD10. Understanding the specific origin of the B-cell leukemia assists in treatment options and prognosis. Craig and Foon ((2008) have described specific immunophenotypic characteristics of different hematologic cancers.

Several nurse-led research projects have used flow cytometry to enhance clinical understanding and to evaluate the physiologic effects of nursing interventions. It was used to evaluate the immune function of geriatric patients who received nutritional supplementation before and after flu shots ((Langkamp-Henken et al., 2006). Flow cytometry also has been used to examine the immune function of caregivers of patients with Alzheimer’s disease ((Thompson et al., 2004). In addition, Motzer and colleagues ((2002) used flow cytometry to examine natural killer and T-cell function in correlation to psychological distress of patients with irritable bowel syndrome. Lastly, since women experience immunosuppression during pregnancy, flow cytometry has been used to examine the immune state of postpartum women over time ((Gennaro et al., 1997).

## Discussion

Flow cytometry has been used for many years in clinical practice. As flow cytometry has advanced, more complex questions and diseases have been diagnosed, explained, and monitored. It is important for APs to have a basic understanding of flow cytometry, since it is a prominent method used in clinical settings. When the AP sees complete white blood cell results, he/she can identify and understand critical values, thereby allowing the AP to anticipate patient needs. The same is true for flow cytometry results. If there is a basic understanding of what is being measured, the AP can anticipate the treatment, understand the implications of the results, and prepare and educate the patient.

Flow cytometry reports in clinical settings, to assist in diagnosis or disease monitoring, usually describe results by means of the actual numbers or percentages of the variable of interest. As previously described, acute myeloid leukemia (AML) and ALL have characteristic subtypes of the disease based on certain morphology, phenotype, and genetics. AML basic precursor cells are phenotypically positive for CD34, CD38, CD117, and HLA-DR (human leukocyte antigen D related). These phenotypic results are usually reported in percentages, with 10% to 20% or greater considered positive markers ((Dohner et al., 2010).

It is crucial to understand that flow cytometry analysis is often used in conjunction with other descriptive tests such as morphologic examination. Pathologists review the cellular morphology of the specimens. Often, hematologic neoplasms depict specific morphologic changes, and flow cytometry provides greater specificity. Flow cytometry often can detect recurrence of cancer before morphologic changes are detected.

Overall, best practices for proper diagnosis should include clinical presentation, morphologic analysis, flow cytometry analysis, and other pertinent testing, such as cytogenetics ((Wood et al., 2007). Limitations to flow cytometry include the facts that the laser can only analyze one cell at a time, cells must be in suspension to be analyzed (thereby restricting the analysis of tissue), highly trained operators are required, and cells must be viable to be analyzed.

Understanding flow cytometry will allow APs to prepare patients and families more effectively for forthcoming diagnoses and treatments. Furthermore, understanding the flow cytometry research involved in a disease or chronic condition, as well as flow cytometry laboratory results, will allow better patient assistance and education.

Revisiting Mrs. K, her bone marrow biopsy results revealed 22.6% CD34⁺/CD117⁺ blasts by flow cytometry, and more detailed flow cytometry and cytogenetic results diagnosed AML. The AP would be able to anticipate the appropriate treatment course for Mrs. K. Counseling and educating patients about AML can begin with both Mrs. K and her family well prepared.
